# Erfolgreicher Einsatz von Anifrolumab bei chronisch diskoidem Lupus erythematodes

**DOI:** 10.1007/s00105-026-05672-8

**Published:** 2026-04-14

**Authors:** Inga Hansen-Abeck, Finn Abeck, Maria Christolouka, Stefan W. Schneider

**Affiliations:** Klinik und Poliklinik für Dermatologie und Venerologie, Universitätsklinikum Hamburg-Eppendorf, Martinistr. 52, 20246 Hamburg, Deutschland

Der systemische Lupus erythematodes (SLE) geht in bis zu 80 % der Fälle mit Hautmanifestationen im Sinne eines kutanen Lupus erythematodes (CLE) einher [1]. Diese treten zumeist in Form eines akuten CLE auf, wobei auch subakute oder chronische Formen des CLE auftreten können [2].

Der primäre CLE ohne systemische Beteiligung manifestiert sich überwiegend als chronisch diskoider Lupus erythematodes (CDLE) [3]. Aufgrund der typischen Lokalisation an sichtbaren Arealen und des hohen Vernarbungsrisikos kann die Erkrankung eine hohe psychische Belastung darstellen [4]. Zur Beurteilung von Krankheitsaktivität und Schaden dient der *Cutaneous Lupus Erythematosus Disease Area and Severity Index* (CLASI), wobei eine Reduktion um 4 Punkte bzw. 20 % als Therapieansprechen gewertet wird [3].

Therapeutisch stehen topische Glukokortikoide und Calcineurininhibitoren sowie verschiedene Systemtherapien im „off-label use“ zur Verfügung [5]. Diese umfassen Hydroxychloroquin als Erstlinientherapie, Methotrexat (MTX), Dapson und Retinoide als Optionen der Zweitlinientherapie sowie Mycophenolat-Mofetil (MMF), Azathioprin, Ciclosporin und Cyclophosphamid in der dritten Therapielinie [5]. Der seit 2012 für den aktiven SLE zugelassene B-Lymphozyten-Stimulator(BLYS)-Antikörper Belimumab kann ebenfalls zu einer signifikanten Verbesserung mukokutaner Manifestationen (insbesondere Exanthem, Alopezie, orale Ulzerationen) führen, die jedoch meist erst verzögert eintritt [5, 6].

Seit 2022 ist der monoklonale Antikörper Anifrolumab als Add-on-Therapie für Erwachsene mit moderatem bis schwerem, aktivem, Antikörper-positivem SLE zugelassen (300 mg intravenös alle 4 Wochen). Die häufigsten Nebenwirkungen sind Infektionen der oberen Atemwege, Infusionsreaktionen und Herpes Zoster. Anifrolumab hemmt selektiv die Aktivität von Typ-1-Interferonrezeptoren (IFNAR1). In einer Post-hoc-Analyse der Zulassungsstudien TULIP‑1 und -2 erreichten 46 % der Patienten mit mukokutaner Beteiligung (initial CLASI-Activity > 10) unter Anifrolumab nach 52 Wochen eine CLASI-Activity-Reduktion von > 50 %, verglichen mit 25 % unter Placebo [7]. Eine Differenzierung zwischen den CLE-Subtypen erfolgte hierbei nicht, sodass diesbezüglich weitere Untersuchungen notwendig sind. Aktuell erfolgt eine Phase-III-Studie, welche den Einsatz von Anifrolumab beim chronischen und subakuten CLE prüft (NCT06015737).

Wir berichten über den erfolgreichen Off-label-Einsatz von Anifrolumab bei Patienten mit CDLE ohne Systembeteiligung.

## Fall 1

**Abb. 1:**
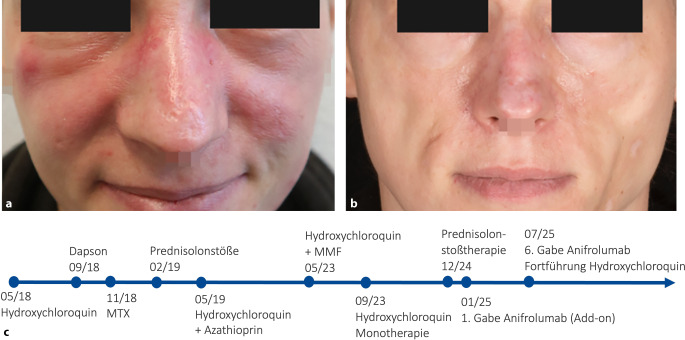
**a** Klinisches Bild vor Beginn von Anifrolumab. **b** Klinisches Bild nach 4 Gaben Anifrolumab mit vollständiger narbiger Abheilung. **c** Chronologischer Verlauf der eingesetzten Systemtherapien. Dosierungen: Hydroxychloroquin 200–400 mg/Tag oral, Dapson 50 mg/Tag oral, Methotrexat (MTX) 10 mg/Woche subkutan, Azathioprin 150 mg/Tag oral, Mycophenolat-Mofetil (MMF) 2000 mg/Tag oral, Anifrolumab 300 mg Q4W intravenös

Eine 38-jährige Patientin entwickelte im Mai 2018 zentrofazial erythematöse Plaques mit Schuppung und zentraler Atrophie (Abb. 1a). Histologisch wurde die Diagnose eines CDLE bestätigt, und ein SLE wurde gemäß den Kriterien des American College of Rheumatology (ACR) und der European Alliance of Associations for Rheumatology (EULAR) ausgeschlossen (Tab. 1).

**Tab. 1:** Charakteristika unserer Patienten

Parameter	Patient 1	Patient 2
Alter bei Erstdiagnose, Geschlecht	38, weiblich	43, männlich
Diagnose	CDLE	CDLE
Lokalisation	Zentrofazial	Bartbereich
Alopezie	Nein	Ja
Schleimhautbeteiligung	Nein	Nein
ANA	1:160	1:320
ENA	Negativ	Negativ
Anti-Phospholipid-Antikörper	Positiv	Negativ
Nikotinabusus	Ja	Nein
ACR/EULAR 2019	6	4
CLASI* vor Anifrolumab	7	5
CLASI* nach Anifrolumab	1	1
Nebenwirkungen unter Anifrolumab	Nein	Nein

* Es wurde der gesamte CLASI (Activity und Damage) erfasst. *ANA* anti-nukleäre Antikörper, *ENA* extrahierbare nukleäre Antigene

Eine topische Behandlung sowie systemische Therapieversuche mit Hydroxychloroquin, Dapson, MTX und Prednisolon sowie MMF blieben erfolglos.

Nach Bewilligung der Kostenübernahme wurde im Januar 2025 eine Add-on-Therapie mit Anifrolumab eingeleitet. Bereits nach der zweiten Infusion zeigte sich ein deutlicher Rückgang der Hautveränderungen sowie des Juckreizes, und innerhalb von 4 Monaten kam es zu einer vollständigen Abheilung aller kutanen Läsionen (Abb. 1b). Nach 6 Gaben wurde die Therapie unter Fortführung von Hydroxychloroquin pausiert, bislang zeigt sich ein stabiler Befund ohne Krankheitsaktivität.

## Fall 2

**Abb. 2:**
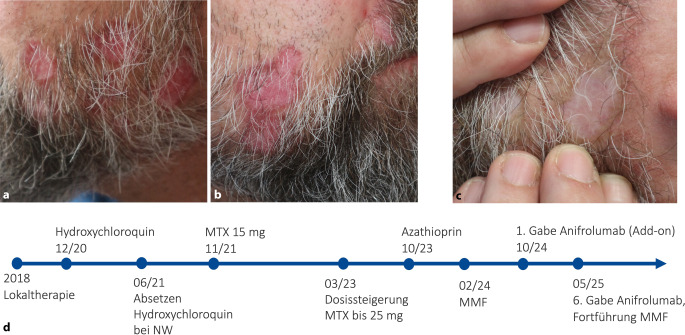
**a** Klinisches Bild vor Beginn von Anifrolumab. **b** Klinisches Bild vor Beginn von Anifrolumab. **c** Klinisches Bild nach 2 Gaben Anifrolumab mit vollständiger Abheilung unter Zurückbleiben einer narbigen Abheilung. **d** Chronologischer Verlauf der Systemtherapien. Dosierungen: Hydroxychloroquin 400 mg/Tag oral, Methotrexat (MTX) 15–25 mg/Woche subkutan, Azathioprin 200 mg/Tag oral, Mycophenolat-Mofetil (MMF) 2000 mg/Tag oral, Anifrolumab 300 mg Q4W intravenös

Ein 43-jähriger Patient stellte sich 2018 aufgrund von stark juckenden, rötlichen Plaques mit Schuppung und einhergehender Alopezie im Bartbereich erstmals vor (Abb. 2a). Nach histologischer Bestätigung und Ausschluss eines SLE (ACR/EULAR 2019: 4 Punkte) wurde die Diagnose eines CDLE gestellt. Nach frustranen Therapieversuchen mit topischen Steroiden und Calcineurininhibitoren sowie multiplen Systemtherapien (Quensyl, MTX, Azathioprin und MMF) wurde im Oktober 2024 eine Add-on-Therapie mit Anifrolumab im „off-label use“ unter Fortführung von MMF begonnen. Bereits nach der ersten Gabe berichtete der Patient von einer wesentlichen Besserung des Juckreizes, und nach 2 Gaben zeigte sich eine komplette Remission des Hautbefundes (Abb. 2b). Nach 6 Infusionen wurde die Therapie beendet und eine Erhaltungstherapie mit MMF fortgeführt. Drei Monate nach Absetzen von Anifrolumab kam es zum Rezidiv, welches unter Re-Einleitung der Add-on-Therapie erneut eine rasche Abheilung zeigte.

## Diskussion

Unsere Fallberichte zeigen ein rasches Ansprechen mit vollständiger Abheilung unter Anifrolumab bei zuvor therapierefraktärem CDLE.

Mehrere Publikationen berichten über eine erfolgreiche Behandlung kutaner Manifestationen bei zugrunde liegendem SLE mit schnellem Ansprechen (zumeist innerhalb von 4 bis 8 Wochen) sowie weitgehend Subtyp-unabhängiger Wirksamkeit sowohl beim chronischen und subakuten CLE als auch bei Patienten mit mukokutaner Beteiligung [8, 9]. Berichte zum Ansprechen bei akuten Formen des CLE fehlen bislang, möglicherweise ist dies auf eine geringere Therapieresistenz dieser Subtypen zurückzuführen.

Der Off-label-Einsatz von Anifrolumab zur Behandlung des therapieresistenten, primären CLE wurde in einem kürzlich veröffentlichten Review für Patienten mit chronischen und subakuten Formen des CLE als wirksam beschrieben [10]. Wie auch bei unseren Fällen wurde eine hohe Effektivität der Therapie mit einem raschen Ansprechen und überwiegend guter Verträglichkeit beobachtet [10].

Es wurde bisher ein Fall berichtet, bei dem es 6 Monate nach dem Absetzen von Anifrolumab zu einem Rezidiv des CLE kam [11]. Ebenso wie in unserem Fall zeigte sich nach Re-Einleitung von Anifrolumab erneut eine gute Wirksamkeit [11].

Anifrolumab greift in die Pathogenese des CLE ein, indem es durch Bindung an IFNAR1 die Signalübertragung blockiert [2]. Dies führt zu einer Reduktion der Expression Typ-I-Interferon-induzierter Gene und in der Folge zu einer reduzierten entzündlichen Aktivität [2]. Es konnte gezeigt werden, dass die Reduktion der Typ-I-Interferon-Gen-Signatur mit dem klinischen Ansprechen korreliert [12]. Der Verlauf nach dem Absetzen wurde bisher noch nicht untersucht, könnte aber für das Verständnis von Rezidiven von Bedeutung sein.

Weitere vielversprechende Therapieoptionen, die derzeit in klinischen Studien beim CLE untersucht werden, sind der Tyrosinkinase 2(TYK2)-Inhibitor Deucravacitinib (NCT04857034, Phase 2) und der Anti-Blood Dendritic Cell Antigen 2(BDCA2)-Antikörper Litifilimab (NCT05531565, Phase 2/3) [13, 14]. Durch die intrazelluläre Blockade von TYK2 werden die Signalwege mehrerer proinflammatorischer Zytokine, darunter Typ-I-Interferone, Interleukin(IL)-12 und IL-23, moduliert, wodurch die Aktivierung dendritischer Zellen sowie von T‑ und B‑Lymphozyten beeinflusst wird [2, 13]. Die selektive Blockade von BDCA2 auf plasmazytoiden dendritischen Zellen reduziert die Produktion von Typ-I-Interferonen und weiteren proentzündlichen Zytokinen an deren zellulärer Quelle [13, 15].

Auf Grundlage unserer Fallberichte sowie der aktuellen Datenlage erscheint Anifrolumab als äußerst vielversprechende Off-label-Therapieoption für Patienten mit primärem CLE, insbesondere bei ausgeprägter Therapieresistenz gegenüber den Standardtherapien.

## Data Availability

Bei berechtigter Nachfrage können die Daten von den Autoren zur Verfügung gestellt werden.
